# lncRNA HITT Inhibits Lactate Production by Repressing PKM2 Oligomerization to Reduce Tumor Growth and Macrophage Polarization

**DOI:** 10.34133/2022/9854904

**Published:** 2022-07-05

**Authors:** Kunming Zhao, Xingwen Wang, Dong Zhao, Qingyu Lin, Yi Zhang, Ying Hu

**Affiliations:** ^1^School of Life Science and Technology, Harbin Institute of Technology, Harbin, Heilongjiang Province, China 150001; ^2^School of Public Health, Qingdao University, Qingdao, China 266071

## Abstract

Lactic acid acidifies the tumor microenvironment and promotes multiple critical oncogenic processes, including immune evasion. Pyruvate kinase M2 (PKM2) is a dominant form of pyruvate kinase (PK) expressed in cancers that plays essential roles in metabolic reprograming and lactate production, rendering it as an attractive therapeutic target of cancer. However, the mechanism underlying PKM2 regulation remains unclear. Here, we show that long noncoding RNA (lncRNA) HIF-1*α* inhibitor at transcription level (HITT) inhibits lactate production in a PKM2-dependent manner. Mechanistically, it physically interacts with PKM2 mapped to a region that has been involved in both dimer (less-active) and tetramer (more-active) formation, inhibiting PKM2 oligomerization and leading to dramatic reduction of PK activity. Under glucose starvation, HITT was reduced as a result of miR-106 induction, which subsequently facilitates PKM2 oligomerization and increases vulnerability to apoptosis under glucose starvation stress. In addition, the interaction also reduces lactate secretion from cancer cells, which subsequently polarizes macrophages toward an M2-like anti-inflammatory phenotype and thus possibly contributes to immune escape *in vivo*. This study highlights an important role of an lncRNA in regulating PKM2 activity and also reveals a metabolic regulatory effect of PKM2 on macrophage polarization.

## 1. Introduction

Cancer is characterized by aerobic glycolysis, known as the Warburg effect, a process where the majority of glucose is metabolized into lactate even in the presence of oxygen [1]. Increased aerobic glycolysis is commonly associated with tumorigenesis and is predictive of metastasis and poor prognosis of patients [2]. The main benefits of aerobic glycolysis are attributed to the production of reducing equivalents and macromolecules to meet the requirements of sustained cancer cell growth, division, and survival [3, 4]. In addition, metabolic reprograming may also alter the tumor microenvironment [5]. For example, although the final product of glycolysis, lactate, used to be considered a waste product of glycolysis, it has been found to play essential roles in the communication between tumor and immune cells, thus contributing to immune escape [6, 7]. It has been reported that increased lactic acid levels acidify the cellular environment and inhibit the activities of immune cells such as macrophages and T cells, leading to tumor cell growth and metastasis [8–10]. In view of the multifaceted functions of metabolic reprograming in cancer, how cancer cells rewire metabolic processes has attracted increased interest and become a major focus of cancer research.

Pyruvate kinase M2 (PKM2), a major cancer-related isoform of pyruvate kinase (PK), has been demonstrated to play a pivotal role in regulating aerobic glycolysis [11], catalyzing the last step of glycolysis to synthesize adenosine triphosphate (ATP) and pyruvate [12]. It has been shown clinically that PKM2 is overexpressed in multiple types of cancer and serves as a predictor of poor prognosis for patients with colon cancer, breast cancer, hepatocellular carcinoma, and many others [13, 14]. Inhibiting PKM2 expression or inactivating its PK activity leads to reduced tumor growth both in vitro and in vivo, further supporting the tumorigenic activity of PKM2 [15–17]. In accordance with this notion, during tumorigenesis, PKM2 levels increase, gradually replacing tissue-specific PK isoforms such as pyruvate kinase 1 (PKM1), until it becomes the dominant isoform [11, 18]. Replacement of PKM2 with PKM1 inhibits tumor formation in nude mouse xenografts [18]. However, paradoxically, PKM1 has greater PK activity than PKM2. If cancer favors low-level PK activity, why does it mainly select for PKM2 overexpression, but not PK inactivation by PKM2 mutation or deletion? A model proposed to explain this discrepancy is that lower levels of PK activity increase glycolytic intermediates upstream of PKM2, which favor biosynthesis and tumor growth, whereas higher-level PK activity may lead to increased generation of ATP to support cell survival under stressful conditions, such as glucose starvation [11, 19–21]. Therefore, tumor cells favor PKM2 because it can switch between a highly active tetramer and a less active dimer form.

Because PK activity dynamics acts as a central node in the regulation of cell growth and survival, understanding how this enzyme is regulated is critical. PKM2 exists as either an inactive monomer, a less-active dimer, or a more-active tetramer [22]. Christofk and colleagues have provided evidence that phosphorylated polypeptides bind with PKM2 and inhibit its allosteric activation, suggesting that PKM2 is subject to regulation by phosphotyrosine signaling stimulated by certain growth factors [23]. Later, several mechanisms have been reported to control the switch between the dimeric and tetrameric forms of PKM2. For example, phosphorylation at Y105 and acetylation at K305 of PKM2 have both been reported to dissociate the tetramer, resulting in reduced PK activity and increased anabolic metabolism and tumor growth [24]. In addition, oncoproteins, such as pp60v-src kinase [25], HPV-16 E7 [26], and A-Raf [27], directly target the dimeric/tetrameric switch of PKM2.

Long noncoding RNA (lncRNA) is a class of transcripts longer than 200 nucleotides (nt) without protein coding potential [28]. Mounting evidence has documented that lncRNAs play vital roles in fundamental biological processes, such as epigenetic, transcriptional, or translational regulation of gene expression and protein degradation or activation [29]. Recently, the involvement of lncRNAs in cancer metabolism has attract great interest [30]. Nonetheless, it remains unknown whether lncRNAs can directly regulate PK activity switch.

We recently identified an lncRNA, namely, HIF-1*α* inhibitor at transcription level (HITT), that is commonly decreased in many types of cancer. Decreased HITT is associated with increased tumor growth and drug resistance [31–33]. Our studies indicate that HITT is a multifunctional lncRNA that produces tumor-suppressive effects by either inhibiting hypoxia inducible factor-1*α* (HIF-1*α*) synthesis-induced angiogenesis or attenuating DNA damage, including ataxia telangiectasia-mutated gene (ATM) activation [31, 33]. HIF-1*α* is also known to be an important regulator of cancer metabolism reprograming [34]. Considering the significance of metabolism in cancer pathology, we wondered whether HITT modulates metabolism. Here, we show that HITT inhibits glycolysis and that, intriguingly, it does so under both normoxic and hypoxic conditions. Further study revealed that, independently of HIF-1*α*, HITT directly binds with PKM2 and blocks its tetramerization. HITT downregulation leads to an increased PK activity, which induces macrophage polarization toward M2-type tumor-associated macrophage via a noncell autonomous mechanism that is dependent on lactate derived from tumor cells.

## 2. Results

### 2.1. lncRNA HITT Inhibits Lactate Production

**Figure 1:**
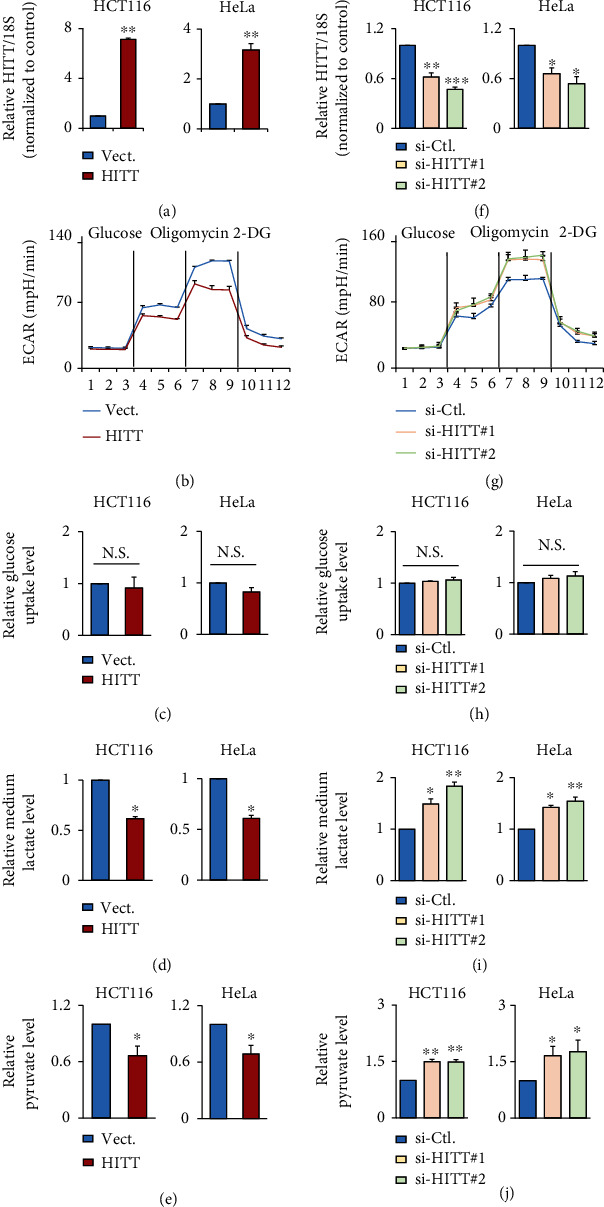
lncRNA HITT inhibits aerobic glycolysis. (a) Overexpression efficiencies of HITT in HCT116 (left) and HeLa (right) stable lines were determined by real-time qRT-PCR. (b–e) ECAR (b), glucose uptake (c), lactate production (d), and pyruvate levels (e) were compared in HITT overexpression and control HCT116 (left) or HeLa (right) cells. (f) The KD efficiency of two independent siRNAs of HITT was confirmed by qRT-PCR, in HCT116 (left) and HeLa (right) cells. HITT expression level was relative to 18S. (g–j) ECAR (g), glucose uptake (h), lactate production (i), and pyruvate levels (j) were compared in HITT KD and control HCT116 (left) or HeLa (right) cells. Data are derived from three independent experiments and presented as mean ± SEM in the bar graphs. Values of controls were normalized to 1. ^∗^*P* < 0.05, ^∗∗^*P* < 0.01, and ^∗∗∗^*P* < 0.001. N.S.: not significant (a, c–f, h–j); Vect.: vector; Ctl.: control.

During culture, we noticed that the color of the media for HITT-overexpressing cells was much pinker than that of controls for multiple types of cancer, such as HeLa, HCT116, HT-29, and H1299 (Figures S1a and S1b). Transient and stable overexpression of HITT produced similar effects (Figures S1a and S1b). These observations led us to ask whether HITT plays roles in modulating cancer cell metabolism per se. To this end, reduced rates of extracellular acidification (ECAR) were compared between control and stable HITT-overexpressing cell lines that had been established previously [31] (Figure 1(a)). The results showed that ECAR was decreased upon feeding glucose and ATP synthase was reduced in HITT-overexpressing cells (Figure 1(b)). Although HITT had no apparent impact on glucose consumption (Figure 1(c)), pyruvate and lactate production was reduced by approximately 30–40% in HITT-overexpressing cells compared with controls (Figures 1(d) and 1(e)). In contrast, two independent small interfering RNA- (siRNA-) mediated HITT knockdown (KD) reduced expression of HITT by about 50% (Figure 1(f)). Accordingly, ECAR and pyruvate and lactate production were increased (Figures 1(g)–1(j)). Recovery HITT expression abolished the effect of HITT KD on lactate production (Figures S1c and S1d). In addition, the ability of HITT to regulate aerobic glycolysis is unlikely cell-type specific, as similar effects of HITT on lactate and pyruvate production were detected in HT-29 and H1299 cells (Figures S1e–S1g). Collectively, HITT inhibits aerobic glycolysis and lactate production.

### 2.2. HITT Inhibits Glycolysis and Lactate Production by Attenuating PK Activity

The next question is what are the mechanisms by which HITT inhibits aerobic glycolysis and lactate production. HIF-1*α* is an essential regulator of cell metabolism [34]. We previously found that HITT inhibits HIF-1*α* mRNA and protein production [31, 32] and reasoned that HITT may regulate glycolysis by inhibiting HIF-1*α* expression; however, it is unlikely because HITT similarly inhibited pyruvate and lactate production in control and HIF-1*α* KD cells under normoxic and hypoxic conditions (Figures S2a–S2d). The color of the media was slightly pink after HITT overexpression regardless of HIF-1*α* expression (Figure S2e).

**Figure 2:**
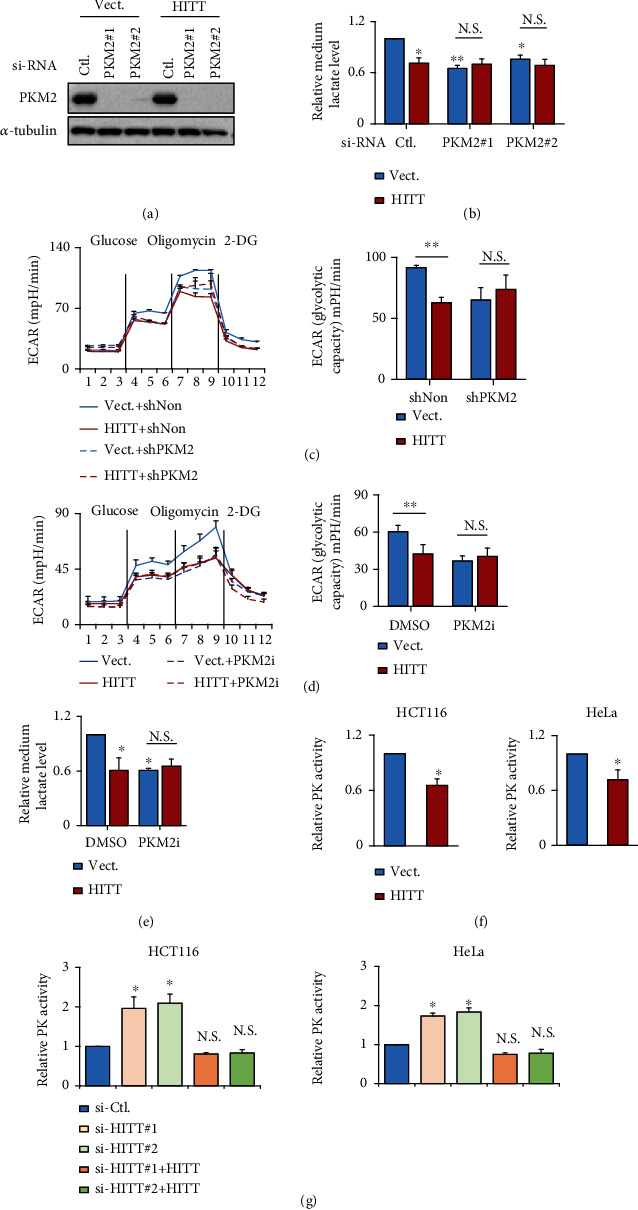
HITT inhibits glycolysis by repressing PK activity. (a) The KD efficiency of PKM2 in the control and HITT-overexpressing stable HeLa cells was confirmed by WB. (b–e) Medium lactate (b, e) and ECAR (c, d) were detected after PKM2 KD or PKM2 inhibitor (PKM2-IN-1, 20 *μ*M for 24 h) treatment. Quantification of the maximal glycolytic capability is shown in the bar graph (right). (f, g) PK activities were determined in HITT-overexpressing (f)/KD with or without HITT recovery (g) HCT116 (left) and HeLa (right) cells. Data are derived from three independent experiments and presented as mean ± SEM in the bar graphs. Values of controls were normalized to 1. ^∗^*P* < 0.05; ^∗∗^*P* < 0.01. N.S.: not significant (b–g); Vect.: vector; Ctl.: control.

We then investigated the potential involvement of key glycolysis enzymes in HITT-regulated metabolism by treating cells with siRNAs specifically targeting individual genes (hexokinase (HK), phosphofructokinase1 (PFK1), or PKM2), which encode the rate-limiting enzymes that regulate glycolysis (Figures S2f and S2g and Figure 2(a)). As expected, silencing of HK, PFK1, or PKM2 reduced lactate levels (Figure S2h and Figure 2(b)). However, neither HK nor PFK1 KD affected HITT's regulation of lactate production (Figure S2h). Intriguingly, PKM2 KD completely abrogated HITT-inhibited glycolysis, as revealed by ECAR and the levels of lactate production (Figures 2(b) and 2(c)), suggesting that PKM2 and HITT produce such activities in the same pathway. Supportively, CRISPR-Cas9-mediated HITT knockout led to an enhanced lactate level, and the effect was also completely diminished by PKM2 KD (Figure S2i). The dependency of HITT on PKM2 was further confirmed in cells pretreated with the PKM2 inhibitor PKM2-IN-1 (Figures 2(d) and 2(e)).

Because the activity of PKM2 is required for HITT to inhibit glycolysis, we asked whether HITT can affect the PK activity of PKM2. To test this, PK activity was evaluated after genetic modulation of HITT expression in HCT116 and HeLa cells. PK activity was consistently reduced with HITT overexpression, while it was increased with HITT KD (Figures 2(f) and 2(g)). Recovery HITT expression abolished the effect on PK activity mediated by HITT KD (Figure 2(g)). In contrast, HITT did not affect the activity of lactate dehydrogenase (LDH), an enzyme that catalyzes the conversion of pyruvate to lactate (Figure S2j). In addition, although nuclear PKM2 has been shown to be essential in regulating glycolysis by modulating gene transcription in the nucleus, it was predominately localized in the cytoplasm, and there was no obvious change in the subcellular distribution of PKM2 in HITT-overexpressing or KD cells (Figure S2k). Therefore, HITT lowers the glycolytic rate and lactate production mainly by inhibiting the PK activity of PKM2.

### 2.3. HITT Acts by Blocking PKM2 Oligomerization

To get insight into the mechanisms involved in HITT-mediated PK activity inhibition, we first examined the impacts of HITT on PKM2 protein levels. However, there were no detectable effects on PKM2 expression at the mRNA and protein levels after HITT overexpression or KD (Figures S3a and S3b).

**Figure 3:**
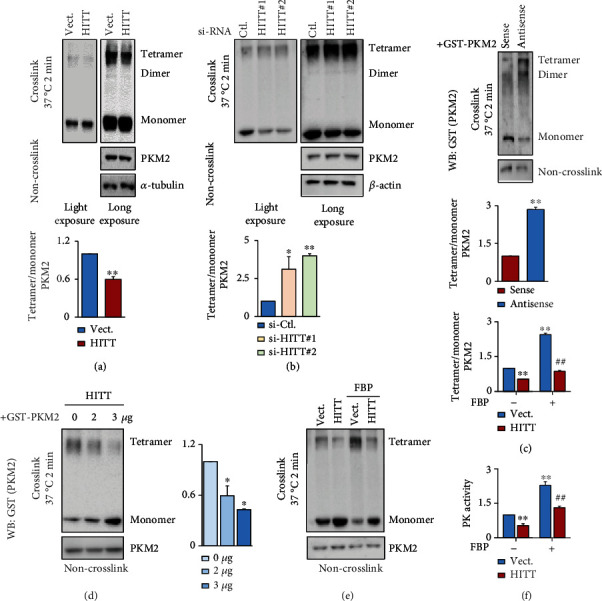
HITT inhibits PKM2 oligomerization. (a, b) The relative expression levels of PKM2 oligomers and monomers were analyzed by WB following glutaraldehyde crosslinking in cell lysates generated from HITT-overexpressing (a) or KD (b) cells. (c) The same amount of purified recombinant GST-PKM2 protein was incubated with 1 *μ*g sense HITT and antisense HITT. After glutaraldehyde crosslinking, the expression levels of PKM2 oligomers and monomers were determined by WB. (d) The same amount of purified recombinant GST-PKM2 protein was incubated with 0, 2, and 3 *μ*g sense HITT. After glutaraldehyde crosslinking, the expression levels of PKM2 oligomers and monomers were determined by WB. (e) The oligomer and monomer distribution of PKM2 was detected after incubation with FBP followed by glutaraldehyde crosslinking. (f) The PK activities of vector and HITT stable cells were detected with or without the addition of FBP (0.2 mM). Data are derived from three independent experiments and presented as mean ± SEM in the bar graphs. Values of controls were normalized to 1. ^∗^*P* < 0.05, ^∗∗^*P* < 0.01 (a–f), and ^##^*P* < 0.01, compared with vector FBP-treated group (e, f). Vect.: vector; Ctl.: control.

It is known that the PKM2 monomer represents the PK-inactive form. PKM2 dimers and tetramers have weak and strong kinase activity, respectively [22]. Therefore, we wondered whether HITT inhibits PKM2 by interfering with its oligomerization. Consequently, PKM2 oligomers, mainly in the form of tetramers, were detected by a glutaraldehyde crosslinking assay. Before glutaraldehyde crosslinking, PKM2 was present as a single band (monomer, bottom lanes), indicating that sodium dodecyl sulfate (SDS) treatment resulted in complete dissociation of potential PKM2 oligomers (Figure 3(a)). After glutaraldehyde crosslinking, the proportion of PKM2 tetramers was robustly increased, which was repressed by HITT overexpression, resulting in a decreased tetramer/monomer ratio (Figure 3(a)). A mild inhibitory effect of HITT on PKM2 dimerization was also detected in long-exposure images. In contrast, HITT KD increased the tetramer/monomer ratio of PKM2 in living cells (Figure 3(b)). To provide more direct evidence, sense or antisense HITT and recombinant PKM2 proteins were synthesized in vitro. Oligomerization of recombinant PKM2 was detected in the absence of HITT by a glutaraldehyde crosslink assay. Then, the same amount of sense or antisense HITT was mixed with recombinant PKM2 protein. It was observed that sense HITT reduced the amount of PKM2 tetramers (Figure 3(c)). However, the same amount of antisense HITT produced no effect on PKM2 tetramerization under the same conditions, suggesting that HITT specifically interferes with the formation of PKM2 tetramers. In line with this idea, PKM2 tetramerization was disrupted by HITT in a dose-dependent manner (Figure 3(d)). FBP treatment improved PKM2 tetramer's formation, and HITT could also lead to a significant decrease in PKM2 tetramer formation and PK activities after incubation with FBP (Figures 3(e) and 3(f)). Therefore, HITT interferes with PKM2 tetramerization, which is in line with the fact that HITT inhibits PKM2 activity.

### 2.4. HITT Is Physically Associated with PKM2 at Its C-Terminus, Mapped to (219–531)

**Figure 4:**
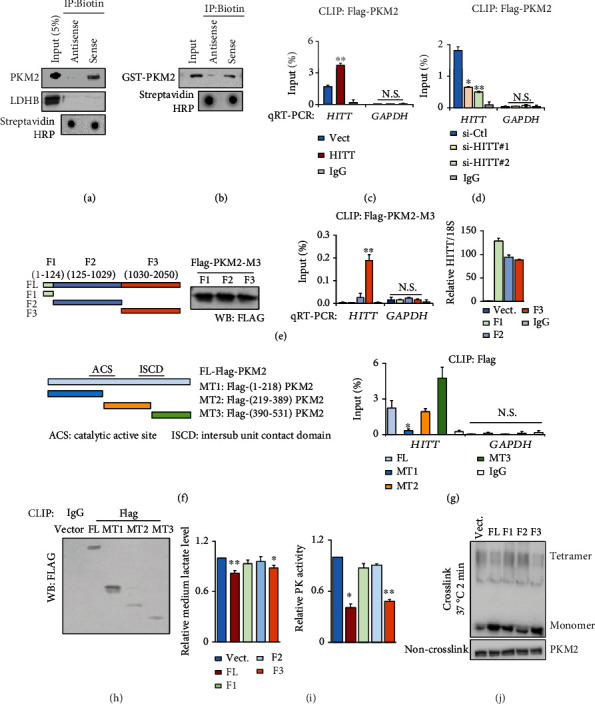
HITT directly binds with PKM2. (a) PKM2 and LDHB levels in protein complexes pulled down by biotin-HITT and biotin-antisense HITT from HeLa cell extracts were detected by WB. (b) Direct interaction between PKM2 and HITT was determined by an in vitro RNA pull-down assay using purified GST-PKM2, in vitro-synthesized biotin-HITT, and biotin-antisense HITT. (c, d) HITT levels of HCT116 cells were determined by real-time RT-PCR following PKM2 CLIP in the control and stable HITT-overexpressing (c) or HITT KD cells (d). IgG and GAPDH RNA were used as controls. (e) A CLIP assay was used to detect the binding of PKM2 with ectopically expressed full-length or HITT fragments as indicated in 4T1 cells (a cell line without endogenous HITT expression). (f–h) HITT levels were determined by real-time RT-PCR following Flag CLIP in HeLa cells after transfection of Flag-tagged full-length (FL) and mutant (MT1–MT3) PKM2, as indicated in the diagram (f). IgG and GAPDH RNA were used as controls (g). The protein pull-down was validated by western blot (h). (i, j) Lactate concentrations and PK activity (i) and oligomers and monomers of PKM2 (j) were analyzed after overexpression of FL or fragmented HITT in HeLa cells. Data are derived from three independent experiments and presented as mean ± SEM in the bar graphs. Values of controls were normalized to 1. ^∗^*P* < 0.05, ^∗∗^*P* < 0.01. N.S.: not significant (c–e, g, i); Vect.: vector; Ctl.: control.

lncRNAs can exert their functions through RNA-protein interactions. To further explain how HITT inhibits PKM2 tetramerization, a biotin RNA-protein pull-down assay was applied to examine the interaction between HITT and PKM2. As shown in Figure 4(a), PKM2 in fresh cell lysate was coprecipitated with biotin-HITT but not biotin-antisense HITT. In contrast, another metabolic enzyme (lactate dehydrogenase B (LDHB)) bound with neither sense HITT nor antisense HITT. In addition, we confirmed that in vitro-synthesized sense HITT, but not antisense HITT, bound with purified recombinant PKM2 protein (Figure 4(b)), suggesting that HITT directly interacts with PKM2. To validate the interaction between HITT and PKM2 in living cells, a UV-crosslinking and immunoprecipitation (CLIP) assay was applied. As shown, the physical interaction between endogenous HITT and PKM2 was detected in cancer cell lines tested (Figures 4(c) and 4(d)). HITT overexpression led to increased binding with PKM2, while HITT KD reduced the binding, leading further evidence to a specific and direct interaction between HITT and PKM2 (Figures 4(c) and 4(d)).

Next, we explored the molecular nature of their interaction. HITT does not have a homolog in mice. To avoid interference from endogenous HITT, we induced expression of similar levels of HITT and HITT fragments in 4T1 cells, a mouse breast cancer cell line (Figure 4(e)). The key residues in HITT were determined by CLIP-FLAG-tagged PKM2. The results showed that the binding ability of HITT fragment 3 (F3) with PKM2 was much more efficient than that of F1 or F2 with the same amount of FLAG-PKM2 protein pull-down (Figure 4(e)). In addition, we determined which domain in PKM2 contributes to the binding with HITT. To this end, mutant-type (MT) PKM2s were generated as indicated in the diagram (Figure 4(f)). Our results revealed that (219–389) MT2 and (390–531) MT3, but not (1–218) MT1, bound with HITT efficiently (Figures 4(g) and 4(h)).

Of note, PKM2 (219–531) has been reported to be essential in PKM2 tetramerization. Intriguingly, only F3, but not other HITT fragments, retained the ability to inhibit subsequent lactate production, PK activity, and tetramerization (Figures 4(i) and 4(j)). As expected, PKM2-dependent regulatory effect on lactate production was not observed when a PKM2 binding defective HITT mutant, F2, was overexpressed (Figure S3c). Therefore, HITT binds with (219–531) PKM2, a region involved in PKM2 tetramerization, via the F3 sequence at (1030–2050) nt and elicits a robust inhibitory effect on PKM2 activity and glycolysis.

### 2.5. HITT Sensitizes Nutrient Stress-Induced Cell Death by Inhibiting PKM2-Dependent Glycolysis

**Figure 5:**
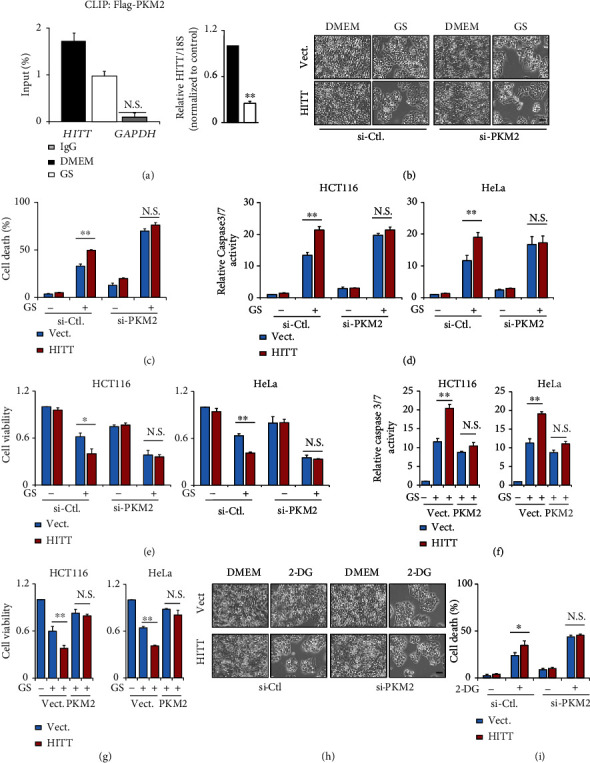
HITT represses adaptive survival under nutrient stress. (a) HITT levels of HeLa cells were determined by real-time RT-PCR following PKM2 CLIP with or without glucose starvation (GS). IgG and GAPDH RNA were used as controls. HITT overexpression efficiency was validated by real-time RT-PCR (right). (b, h) Representative phase contrast images of HeLa cells after PKM2 KD and/or glucose starvation (b) or 2-DG (h) treatment are presented. Scale bar, 100 *μ*m. (c, i) The cell death rates were determined by the trypan blue exclusion assay after PKM2 KD and/or glucose starvation (c) or 2-DG (i) treatment, in HeLa cells. (d, f) The caspase-3/7 activities in HITT overexpression HCT116 and HeLa cells were detected after PKM2 KD (d) or PKM2 overexpression (f) treated with glucose starvation. (e, g) The cell viability of HITT-overexpressing HCT116 and HeLa cells was analyzed after PKM2 KD (e) or PKM2 overexpression (g) treated with glucose starvation. Data are derived from three independent experiments and presented as mean ± SEM in the bar graphs. Values of controls were normalized to 1. ^∗^*P* < 0.05, ^∗∗^*P* < 0.01. N.S.: not significant (a, c–g, i); Vect.: vector; Ctl.: control.

Metabolic reprograming is required for cell survival under nutrient stress, which frequently occurs during tumorigenesis. Considering its critical roles in modulating glycolysis, we asked whether HITT regulates cell adaptive survival under conditions of glucose starvation. To this end, cells were treated with glucose-free medium. Intriguingly, HITT expression was dramatically reduced upon glucose starvation, which was accompanied by reduced HITT and PKM2 interaction (Figure 5(a)); siRNA-mediated PKM2 inhibition induced glucose starvation-induced cell death (Figures 5(b)–5(e)), while PKM2 overexpression improved the adaptive survival of cancer cells under glucose starvation (Figures 5(f) and 5(g)). When HITT expression was reactivated under glucose starvation conditions, cell viability was dramatically reduced (Figures 5(b)–5(g)), while cell proliferation rate, as indicated by BrdU incorporation, was not changed (Figures S3d and S3e). However, this ability of HITT was completely abolished in PKM2 KD (Figures 5(b)–5(e)) or PKM2-overexpressing cells (Figures 5(f) and 5(g)). These data suggest that HITT-inhibited PKM2 is essential to promote glucose starvation-mediated cell death. This effect of HITT was also validated in the presence of a glycolysis inhibitor, deoxyglucose (2-DG, Figures 5(h) and 5(i)).

### 2.6. miR-106 Contributes to the Decreased HITT Expression under Nutrient Stress

**Figure 6:**
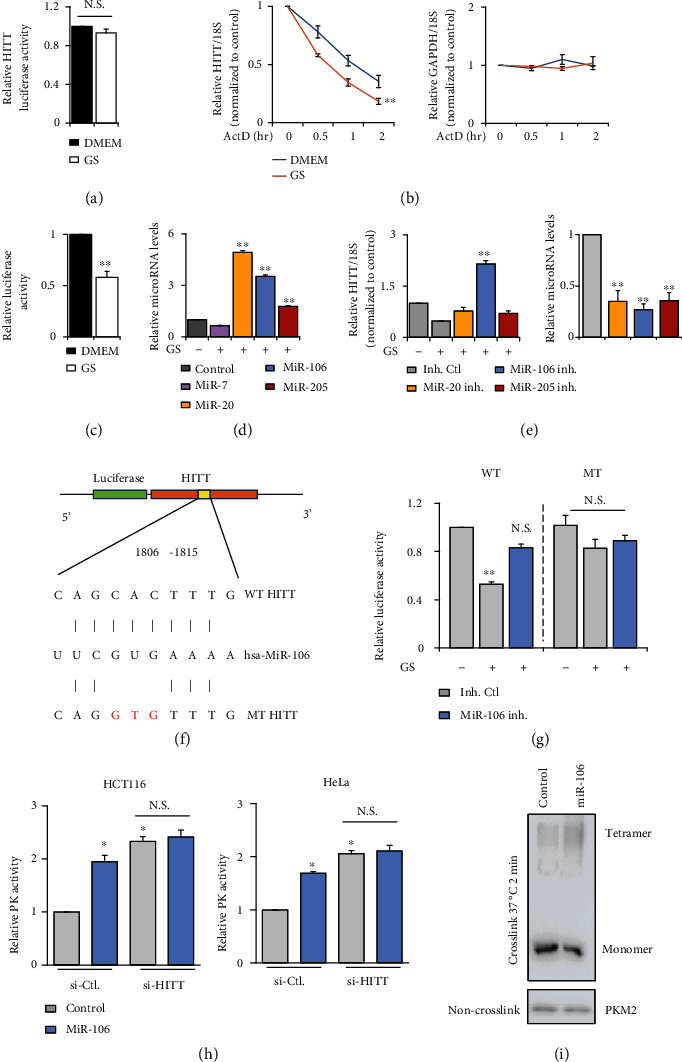
HITT was reduced upon glucose starvation through miR-106. (a) HITT promoter-driven luciferase activity was detected by the luciferase reporter assay under GS in HeLa cells. (b) The half-lives of HITT and GAPDH mRNA were measured by qRT-PCR in the presence of ActD in HeLa cells with or without GS. (c) The luciferase activities of the pMIR-HITT reporter were detected in HeLa cells with or without GS. (d) Expression levels of microRNA normalized to U6 were measured by qRT-PCR under GS in HeLa cells. (e) Relative expression levels of HITT were determined by qRT-PCR after transfection with microRNA inhibitors (inh.) in HeLa cells. MicroRNA levels normalized to U6 were measured by qRT-PCR. (f) Schematic description of the hypothetical duplexes formed by interactions between the binding site in HITT (top), miR-106 (middle), and the mutated HITT (bottom). (g) The luciferase activities of wild-type (WT) or miR-106 binding defective mutant (MT) HITT luciferase reporter were detected in HeLa cells after transfection with the miR-106 inhibitor with or without GS, as indicated in the figures. (h) PK activities of HCT116 and HeLa were detected after transfection of miR-106 with or without HITT KD. (i) The expression levels of PKM2 monomers and oligomers were determined after miR-106 transfection. Data are derived from three independent experiments and presented as mean ± SEM in the bar graphs. Values of controls were normalized to 1. ^∗^*P* < 0.05, ^∗∗^*P* < 0.01 (c–e, g, h). N.S.: not significant (a–e, g, h); Ctl.: control.

Considering that HITT downregulation facilitates cancer cells to adapt to glucose starvation, we next explored how HITT levels are decreased upon glucose starvation. Our results showed that HITT promoter-driven luciferase activity has no obvious changes in the control and glucose-starved cells (Figure 6(a)). The RNA synthesis inhibitor actinomycin D (Act D) reduced HITT levels as expected, whereas it declined quicker in glucose-starved cells than in the controls. In contrast, Glyceraldehyde-3-Phosphate Dehydrogenase (GAPDH) mRNA control was not changed with glucose starvation or HITT expression (Figure 6(b)). These data suggest that HITT is reduced by promoting RNA decoy under glucose starvation. To test this hypothesis, we generated a reporter with the HITT sequence cloned downstream of the luciferase reporter, namely, pMIR-HITT reporter. Supportively, the luciferase activity of this reporter was found to be significantly decreased upon glucose starvation (Figure 6(c)). These data collectively suggest that glucose starvation reduced HITT stability.

MicroRNAs play essential roles in reducing not only mRNA stability but also those of lncRNAs [35]. We previously identified four microRNAs, miR-205, miR-106, miR-7, and miR-20, which can inhibit HITT expression [31]. Here, we found that HITT decoy is mainly due to the expression of miR-106. First of all, miR-106 was upregulated by glucose starvation and exhibited a negative association with HITT levels (Figure 6(d)). Secondly, diminishing miR-106 expression by miR-106 inhibitors abolished glucose starvation-mediated HITT downregulation (Figure 6(e)). Thirdly, the pMIR-HITT reporter mutated at miR-106 binding sites, generated by mutagenesis kits (Figure 6(f)), failed to respond to glucose starvation or miR-106 inhibitor (Figure 6(g)). miR-106 enhanced PKM2 catalysis activity, but this ability was restricted after HITT KD (Figure 6(h)). miR-106 also remarkably increased PKM2 tetramerization (Figure 6(i)). In addition, we analyzed the correlation between the expression of miR-106 and HITT from the ENCORI Pan-Cancer Analysis Platform (https://starbase.sysu.edu.cn/panCancer.php); miR-106 has a significantly negative correlation with HITT in colon adenocarcinoma (Figure S4). This result derived from clinical data demonstrates a correlation between miR-106 and HITT in vivo. Collectively, miR-106 upregulation leads to a decreased HITT expression, which promotes PKM2 catalysis activity and antagonizes the adaptive survival of cancer cells under glucose starvation.

### 2.7. HITT Inhibits Macrophage Polarization by Reducing PKM2-Dependent Lactate Generation

**Figure 7:**
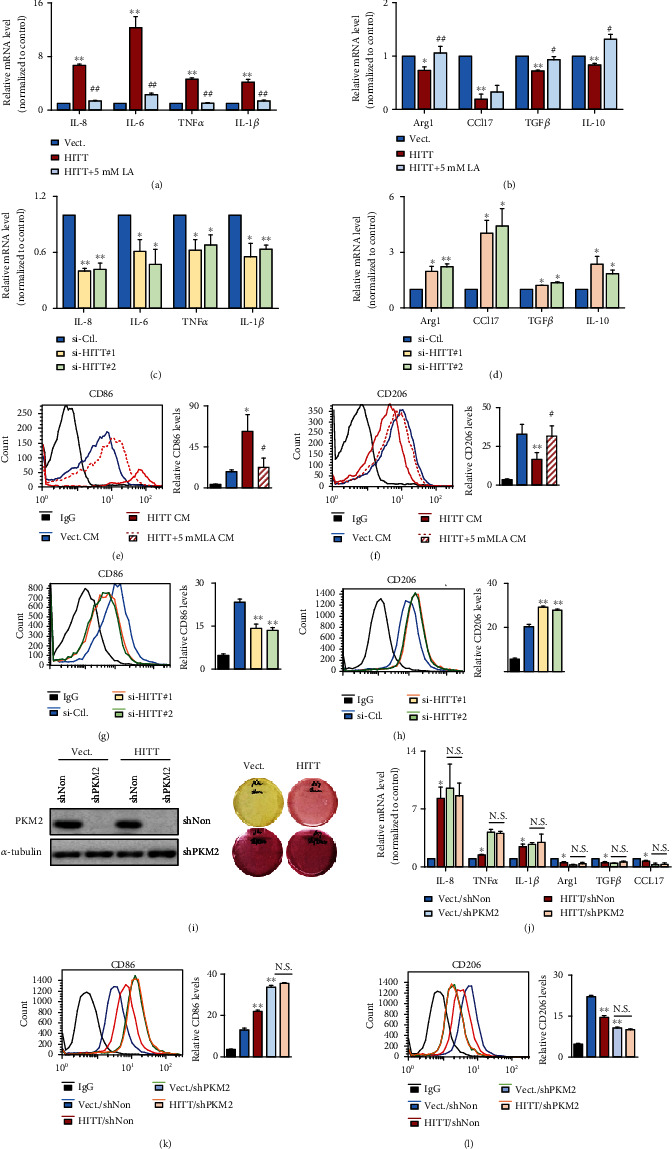
HITT-regulated PKM2-lactate repression alleviates M1–M2 macrophage polarization. (a–d) The mRNA expression levels of IL-8, IL-6, TNF*α*, IL-1*β*, and INOS (a, c) or Arg1, CCL17, TGF*β*, and IL-10 (b, d) were analyzed by real-time RT-PCR in THP-1 cells cultured with conditioned medium (CM) from HeLa cells after the indicated treatments. (e–h) The CD86 (e, g) or CD206 (f, h) expression levels of THP-1 cells cultured with CM from HeLa cells after the indicated treatments were determined by flow cytometry. (i) PKM2 KD efficiencies of the indicated stable cells were determined by WB (left). Right photos show the representative media colors of these stable cells. (j) The mRNA expression levels of IL-8, TNF*α*, IL-1*β*, Arg1, TGF*β*, and CCL17 were analyzed by real-time RT-PCR in THP-1 cells cultured with conditioned medium (CM) from HeLa cells after the indicated treatments. (k, l) The CD86 (k) or CD206 (l) expression levels of THP-1 with conditioned medium (CM) from HeLa cells after the indicated treatments were determined by flow cytometry. Data are derived from three independent experiments and presented as mean ± SEM in the bar graphs. ^∗^*P* < 0.05, ^∗∗^*P* < 0.01. N.S.: not significant (a–h, j–l). ^#^*P* < 0.05, ^##^*P* < 0.01, compared with HITT CM-treated group (a, b, e, f). Vect.: vector; Ctl.: control; LA: lactic acid.

In addition to the autonomous effect of HITT on cell viability, we also speculate that HITT acts in a nonautonomous manner to skew immune microenvironment, because one important finding of this study was the dramatic change in lactate levels after HITT expression and PKM2 inhibition. It has been shown that lactate production by tumor cells plays an important role in macrophage polarization toward an M2-like phenotype [10]. Therefore, we further explored HITT's function in macrophage polarization. To do this, human leukemia monocyte THP-1 cells were triggered to be differentiated to a macrophage by 12-O-tetradecanoylphorbol-l3-acetate (PMA). The expression of representative biomarkers for M1- and M2-like macrophages was examined in differentiated THP-1 cells after culture with conditioned medium (CM) derived from HeLa cells. M1-like macrophage markers such as interleukin-6 (IL-6), interleukin-8 (IL-8), tumor necrosis factor-*α* (TNF*α*), and interleukin-1*β* (IL-1*β*) were dramatically induced by CM from stable HITT-overexpressing cells (Figure 7(a)). M2 markers of macrophages, such as arginase 1 (Arg1), C-C motif chemokine ligand 17 (CCL17), transforming growth factor *β* (TGF*β*), and interleukin-10 (IL-10), were decreased by CM from HITT-overexpressing HeLa cells (Figure 7(b)). In contrast to HITT overexpression, CM derived from HITT KD HeLa cells produced the opposite effect on macrophage polarization (Figures 7(c) and 7(d)). To balance the difference of lactate level between the conditional medium of control (12 mM) and HITT overexpression (7 mM) groups, additional lactic acid was added into HITT CM. Remarkably, such an effect of HITT was largely compromised by balancing the lactate level in HITT CM to that of the control cells (Figures 7(a) and 7(b)).

To provide further evidence for a role of HITT-lactate inhibition in macrophage polarization, levels of cell surface markers CD86 and CD206, which are M1 and M2 markers, respectively, in THP-1 cells were examined by flow cytometry (FACS). Consistently, HITT CM promoted CD86 and repressed CD206 expression levels in THP-1 cells, and this was also lactate-dependent (Figures 7(e) and 7(f)). In contrast, CM from HITT KD cells produced the opposite effect on the expression of CD86 and CD206 in THP-1 cells (Figures 7(g) and 7(h)). Further, the effects of HITT CM on macrophage polarization were completely abrogated by PKM2 KD (Figures 7(i)–7(l)). Therefore, our data collectively lead to the notion that HITT promotes macrophage polarization to M1 by inhibiting PKM2 activity and subsequent lactate production into the environment.

Furthermore, it has been shown that lactate can inhibit macrophage infiltration. Indeed, our data revealed that lactate promoted macrophage infiltration in a dose-dependent manner (Figures S5a and S5b). However, HITT-regulated lactate inhibition was not sufficient to generate a dramatic effect on macrophage infiltration under our experimental conditions (Figures S5c and S5d). We consistently observed an inhibitory effect of HITT on macrophage infiltration in a lactate-dependent manner, while no statistical significance was obtained by analyzing three independent experiments (Figures S5c and S5d).

Therefore, HITT CM regulates macrophage activity mainly by influencing its polarization.

### 2.8. HITT-PKM2-M2 Polarization Confers Reduced Tumor Growth In Vivo

**Figure 8:**
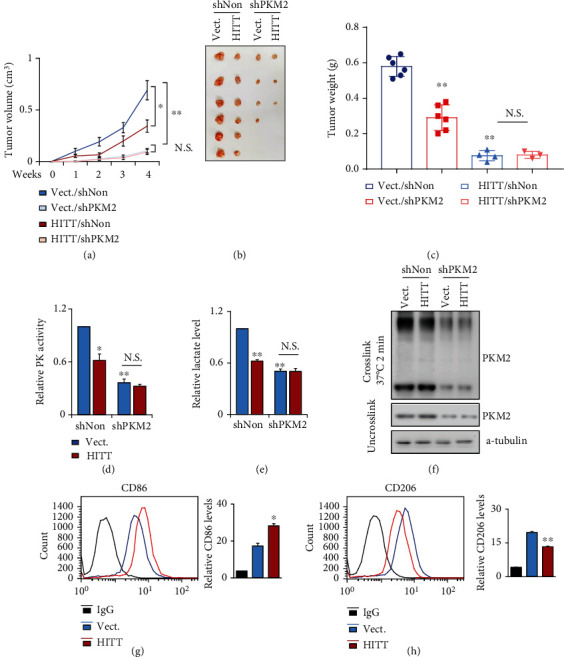
HITT-regulated PKM2-lactate repression inhibits tumor growth in vivo. (a–c) Tumor volumes at the indicated dates (a), as well as images (b) and tumor weights (c) at 4 weeks, for HCT116/vector (Vect.+shNon), HCT116/HITT (HITT+shNon), HCT116/(Vect.+shPKM2), and HCT116/(HITT+shPKM2) xenografts. The average values are presented as bar graphs (means ± SD) (*n* = 6 for each group). (d–f) PK activities (d), lactate levels (e), and PKM2 tetramers (f) were detected in the tumor tissues of xenografts. (g, h) The CD86 (g) or CD206 (h) expression levels of macrophages from tumor tissues of xenografts were determined by flow cytometry. Data are derived from three independent experiments and presented as mean ± SD in the bar graphs. ^∗^*P* < 0.05, ^∗∗^*P* < 0.01. N.S.: not significant (a, c-e, g, h); Vect.: vector.

To determine whether HITT-mediated PKM2 inhibition and subsequent lactate production contribute to tumor growth in vivo, a xenograft model with different HITT or PKM expression levels was applied. After the establishment of stable HCT116 sublines with different expression levels of HITT and PKM2, the same numbers of cells were inoculated subcutaneously in nude mice. The growth rates were monitored, and the results are shown in Figure 8(a). HITT overexpression repressed tumor growth. A similar inhibitory effect was detected in PKM2 KD xenografts; however, no further reduction was detected with the combination (Figure 8(a)). Five weeks posttransplant, mice were euthanized and the xenografts were dissected and weighed (Figures 8(b) and 8(c)). In line with the tumor growth curve, we found that HITT overexpression and PKM2 KD repressed tumor weight, while the effects of HITT were largely diminished in PKM2 KD xenografts (Figures 8(a)–8(c)).

In addition, PK activity, lactate production, and tetramer formation of PKM2 were reduced by HITT overexpression or PKM2 KD, and no further reduction was observed with the combination in vivo (Figures 8(d)–8(f)). Macrophage polarization was also examined in the indicated xenografts. In line with the results obtained in vitro, M1-like polarization was evident after HITT overexpression, as indicated by CD86 and CD206 (Figures 8(g) and 8(h)).

## 3. Discussion

Aerobic glycolysis is a newly identified hallmark of cancer [5]. The expressions of several enzymes have been found to be overexpressed in different tumor types and have been shown to be required for tumorigenesis [36]. PKM2 is one of the cancer-specific glycolysis enzymes and is thus as an attractive target for cancer therapy [36, 37]. PKM2 regulatory mechanisms that are expected to point to distinctive strategies to target PKM2 have emerged as attractive topics in cancer research.

Here, we present a new mechanism that cancer cells utilize to inhibit PKM2 activity by an lncRNA, HITT. It has been reported previously that posttranslational modification and oncoprotein-mediated interaction can disrupt tetramer formation, leading to an increased dimer/tetramer ratio [22]. As the tetramer has relatively higher PK activity than the dimer, such regulation leads to reduced PK activity. Intriguingly, we found that HITT's direct binding with PKM2 maps to a region that involves the essential residues for both dimer and tetramer formations. It is not surprising that HITT interferes with both PKM2 dimerization and tetramerization. This is interesting, because it has been proposed that low-activity PKM2 (dimer) leads to the accumulation of building blocks (metabolic intermediates) that meet the requirements of the fast-proliferating cancer cells, whereas high-activity PKM2 (tetramer) favors bioenergetic production that may be essential for the survival of cancer cells under stress conditions, such as nutrient (glucose) starvation [12, 19–21]. It logically flows that if HITT inhibits both functionalities of PKM2, HITT may be more potent than previously reported allosteric regulators of PKMs in inhibiting carcinogenesis. In accordance with this idea, HITT strongly inhibits PKM2 activity. Consequently, it reduces adaptive cell survival under glucose starvation and inhibits tumor growth in vivo. However, it should also be noted that low abundance of lncRNA produces dramatic effect on PKM2 activation. Whether additional factors are subsequently involved following the initial binding of HITT with PKM2? If so, how such factors ensure PKM2 in an inactive format, even when HITT is released from PKM2 binding? Or whether phase separation provides additional layer of HITT-mediated PKM2 inhibition? All these questions needed to be answered in the future.

Recently, a number of lncRNAs have been suggested to play essential roles in regulating PKM2, in addition to HITT. However, these lncRNAs mainly act by modulating PKM2 protein abundance, while the discovery of HITT points to an alternative that involves the regulation of PKM2 activity. In addition, we revealed that PKM2 has RNA-binding activity. In agreement with our data, Bian et al. recently demonstrated that lncRNA-FEZF1-AS1 also directly interacts with PKM2 [15]. We mapped the HITT binding sites to (219–531) PKM2, which overlaps with the region that contributes to the binding with lncRNA-FEZF1-AS1, PKM2 (219–350). However, Bian et al. also reported that lncRNA-FEZF1-AS1 induces PKM2 protein degradation after binding, while the binding of HITT with PKM2 has no apparent effect on PKM2 protein levels. The mechanisms of lncRNA-PKM2 interactions at overlapping regions may be distinct.

Also notably, HITT was initially identified as an inhibitor of HIF-1*α*, which is also an important regulator of metabolism [34]. Although we found that HITT regulated glycolysis under normoxia independently of HIF-1*α*, HITT-inhibited tumor growth in vivo may be at least partially attributed to the activation of HIF-1*α* in xenografts. That is because HIF-1*α* expression is easily detected in xenografts. Reduced HIF-1*α* expression is evident in HITT-overexpressing xenografts [31]. This is in line with our data that PKM2 KD largely diminishes, but does not abolish, the effect of HITT in xenograft growth. Our data suggest that HIF-1*α* and PKM2 are both essential downstream factors for HITT's inhibition of tumorigenesis, while the proportional contribution of HIF-1*α* and PKM2 to HITT-mediated tumor suppression in vivo needs to be evaluated in future studies, and whether HITT may bind with additional glycolysis regulators under different conditions warrants further investigation.

In metabolism, pyruvate synthesized by PKM2 can be further converted to either lactate by LDH or acetyl-CoA in the mitochondria to fuel oxidative phosphorylation [38]. However, in the context of aerobic glycolysis, the majority of pyruvate is converted to lactate rather than acetyl-CoA [1, 39]. Acidity is a hallmark of the cancer milieu [6]. Lactate has been shown to promote tumor growth and metastasis and is also often associated with poor prognosis [40]. Of interest, lactate has been recently identified as a signaling molecule that is involved in the interaction between tumors and microenvironment immune cells by polarizing macrophages toward an M2-like phenotype, consequently conferring immune escape [10, 41]. Tumor-associated macrophages often exhibit an anti-inflammatory phenotype, playing roles in driving tumor cell growth and metastasis [42]. The consistent and evident changes of lactate secretion into the culture medium after modulating the HITT-PKM2 axis inspired us to investigate whether HITT-PKM2-lactate plays roles in regulating macrophage polarization. Indeed, HITT-PKM2 polarizes macrophages toward an M2-like phenotype. This is lactate-dependent, because adding lactate back to the medium of HITT KD cells abolished its effect on M2 polarization. HITT-PKM2-regulated M2 polarization was also validated in xenografts. These data suggest that HITT-PKM2-regulated tumor growth may be at least partially attributed to lactate-mediated macrophage polarization. Notably, immune cells use glycolysis as their main energy source [43]. It is thus not surprising that PKM2 activation is an essential factor in the autonomous regulation of immune cell activity. Our data provide another layer of regulation of PKM2 in the immune response via a noncell autonomous mechanism elicited by lactate derived from tumor cells. It is thus interesting to propose that PKM2 inhibition may not only inhibit tumor growth but also could sensitize cells to immune therapies. This idea is worthy of future testing.

**Figure 9:**
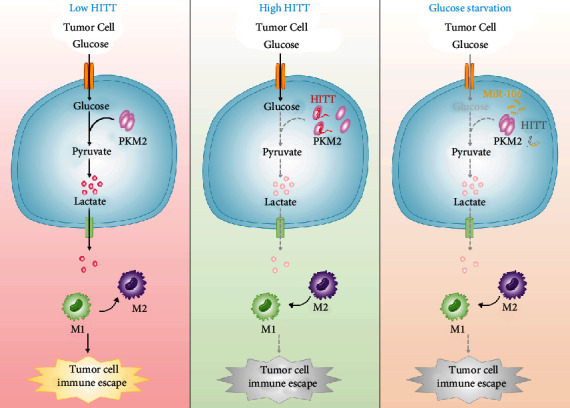
HITT-regulated PKM2-lactate repression inhibits tumor growth in vivo. Pyruvate kinase M2 (PKM2) plays essential roles in metabolic reprograming and lactate production. The tetramer formation of PKM2 has more active PK activity and regulates M2 polarization of macrophages via lactate derived from tumor cells. Under glucose starvation, miR-106 is increased, which inhibits lncRNA HITT levels and facilitates PKM activation and adaptive survival. This is because HITT inhibits PKM2 dimer and tetramer formation and subsequent lactate production into the environment via direct interaction. So HITT inhibits tumor growth by inhibiting PKM2 activity and promoting macrophage polarization to M1.

In conclusion, we identified a new mechanism that inhibits PKM2 dimer and tetramer formation and consequently its activity, via the lncRNA HITT. HITT-PKM2 inhibits tumor growth and regulates M2 polarization via lactate derived from tumor cells (Figure 9). These findings open up new avenues for the manipulation of cancer metabolism and PKM2 activation and may provide ways to target this fundamental process.

## 4. Materials and Methods

### 4.1. Cell Culture and Chemicals

HCT116 and THP-1 (Guangzhou Cellcook Biotech Co., Ltd.) cells were cultured in RPMI-1640 medium (Gibco, Carlsbad, CA, USA) supplemented with 10% (*v*/*v*) fetal bovine serum (Biological Industrial). HeLa, HT-29, HEK-293, and 4T-1 cells were grown in Dulbecco's modified Eagle's medium (DMEM) (Gibco, Carlsbad, CA, USA) with the same supplements. HITT overexpression stable HeLa and HCT116 cells were established previously. All cells were grown in the humidified incubator (Thermo Scientific) with 5% CO_2_. Cells used before experiments were tested to avoid mycoplasma contamination.

Critical chemicals used in this study were shown as follows: PMA (Selleck, S7791), PKM2-IN-1(MedChemExpress, HY-103617), glucose (Sigma, G7528), oligomycin (Apexbio, C3007), 2-DG (Apexbio, B1027), D-lactic acid (Sigma, L6402-1G), and FCCP (MedChemExpress, HY-100410).

### 4.2. Short Hairpin (sh) RNA Constructs and Lentiviral Production

The shRNA target sequence for PKM2 and control were 5′-CCGGGCTGTGGCTCTAGACACTAAACTCGAGTTTAGTGTCTAGAGCCACAGCTTTTTG-3′ and 5′-CCGGGAGGCTTCTTATAAGTGTTTACTCGAGTAAACACTTATAAGAAGCCTCTTTTTG-3′, which were subcloned into a PLKO.1-GFP lentiviral plasmid vector [44]. Lentivirus was packaged with HEK293T cells using a two-plasmid system. Briefly, control shRNA (shNon) or shPKM2 plasmid was cotransfected with pCMV.△8.9 and VSV-G plasmids. Lentivirus secreted in the medium was collected 48 h after transfection and ready to infect target cells in the presence of polybrene (10 *μ*g/ml). 72 h after infection, single clones were selected by limited dilution.

### 4.3. siRNA and Plasmids

Nonspecific si-scramble control or siRNA specifically targeting HITT, PKM2, and HIF-1*α* was transfected into cells by Lipofectamine 2000 by following the manufacturer's instruction. Cells were ready for the subsequent analysis 72 h after transfection. siRNA oligos used for targeting HITT were the same as those reported previously [31]. The siRNA oligo sequences used to target PKM2 and HIF-1*α* are listed as follows: si-PKM2#1(CCAUAAUCGUCCUCACCAA), si-PKM2#2(UUGGUGAGGACGAUUAUGG), si-HIF-1*α*#1(CCAGCAGACUCAAAUACAATT), si-HIF-1*α*#2(GCAGCUACUACAUCACUUUTT), si-HK2#1(CCGTAACATTCTCATCGATTT), si-HK2#2(ACTGAGTTTGACCAGGAGATT), si-PFK#1(CCTCCAGAAAGCAGGTAAGAT), and si-PFK#2(CACTCAATACTATCTGCACAA), which were synthesized by GenePharma (Shanghai, China).

The full-length PKM2 were kindly provided by Prof. Qunying Lei, Fudan University [44]. The indicated PKM2 mutants were subcloned into a pcDNA3.1-3xFlag or pGEX-6p-1 vector in this study. WT or miR-106 binding site mutant (MT) HITT was inserted into the PMIR-reporter downstream of luciferase element, namely, WT HITT reporter and MT HITT reporter, respectively. The CRISPR/Cas9-*HITT* plasmid was constructed as in our previous protocol [45]. The sequences of two gRNAs targeting *HITT* in the modified px458 plasmid are listed as follows: forward 5′-GAGGGGCACGGTAACACC-3′ and downstream 5′-TGCCAGACGGGTCGGGTG-3′.

### 4.4. RNA Extraction and Real-Time PCR

TriZol reagent (Invitrogen, Carlsbad, CA, USA) was used to isolate total RNA by following the manufacturer's protocol. cDNA was synthesized by using a PrimeScript reverse transcription (RT) (Takara, #RR047A) reagent kit with gDNA Eraser, followed by qRT-PCR analysis using a SYBR Premix Ex Taq II kit (Takara, #RR820L) in the ViiA7 real-time PCR (Applied Biosystems) system. The primers were synthesized by Comate Bioscience (Changchun, China). Sequences of primers were shown as follows: HITT F5′-ACACAAATGCTGGCCTCTGTCA-3′ and R5′-GGCAAGTGGCAAAGCCTCTC-3′, PKM2 F5′-GATGGAGCCGACTGCATCATG-3′ and R5′-TCTGTGGGGTCGCTGGTAATG-3′, IL-6 R5′-ACAGCCACTCACCTCTTCAGAACG-3′ and R5′-CCAGGCAAGTCTCCTCATTGAATCC-3′, IL-8 R5′-ACATACTCCAAACCTTTCCACCC-3′ and R5′-TTCTCAGCCCTCTTCAAAAACTTC-3′, IL-10 F5′-GACTTTAAGGGTTACCTGGGTTG-3′ and R5′-TCACATGCGCCTTGATGTCTG-3′, TNF*α* F5′-CCTCTCTCTAATCAGCCCTCTG-3′ and R5′-GAGGACCTGGGAGTAGATGAG-3′, IL-1*β* F5′-AAAGCCATAAAAACAGCGAGGG-3′ and 5′-TGGTGGTCGGAGATTCGTAG-3′, Arg1 F5′-ACGGAAGAATCAGCCTGGTG-3′ and R5′-ATCAGTGTGAGCATCCACCC-3′, CCL17 forward 5′-TTCTCTGCAGCACATCCACG-3′ and R5′-AAACGATGGCATCCCTGGAG-3′, TGF*β* forward 5′-GGAAACCCACAACGAAATCTATGAC-3′ and R5′-GCTGAGGTATCGCCAGGAATT-3′, 18s F5′-AACTTTCGATGGTAGTCGCCG-3′ and reverse 5′-CCTTGGATGTGGTAGCCGTTT-3′, and GAPDH F5′-TCGTCTGAGGGGACAGGAGGATC-3′ and R5′-GGAAAGGCAAGTCCAGAGGTGGG-3′.

### 4.5. Western Blot Assay

Urea buffer (8 M urea, 1 M thiourea, 0.5% CHAPS, 50 mM DTT, and 24 mM spermine) was used to isolate total protein. The equal amount proteins were separated by SDS-PAGE. The indicated primary antibodies and the secondary antibodies were applied to the PVDF membrane with proteins, and the signal was visualized by using an ECL kit (Thermo Scientific, #32106). Antibodies used for western blot (WB) and the corresponding dilution rates were listed as follows: PKM2 (Proteintech, 1 : 1000, 15822-1-AP), HIF-1*α* (Abcam, 1 : 2000, ab51608), *β*-actin (Proteintech, 1 : 2000, 60008-1-Ig), LDHB (Proteintech, 1 : 1000, 14824-1-AP), FLAG (Proteintech, 1 : 2000, 20543-1-AP), *α*-tubulin (Proteintech, 1 : 2000, 66031-1-Ig), GST (ABclonal, 1 : 2000, AE006), and Histone 3.1 (#KM9005T, 1 : 2000, Sungene).

### 4.6. UV-Crosslinking RNA-IP (CLIP)

Cells irradiated with UV were collected in lysis buffer (5 mM PIPES (pH 8.0), 85 mM KCl, 0.5% NP40, 1% SDS, 10 mM EDTA, and 50 mM Tris-HCl, pH (8.1)), supplemented with inhibitors (Thermo Fisher). Protein G sepharose beads were incubated with lysate for 1 h, followed by the incubation with antibodies or immunoglobulin G (IgG) control, rotating at 4° C overnight for at least 20 h. The RNA from the antibody-protein-RNA complexes was isolated and used for further qRT-PCR analysis.

### 4.7. In Vitro RNA Pull-Down Assay

Biotin RNA Labeling Mix (Roche, 11685597910) was used to synthesize biotin-labeled HITT and its antisense in vitro. DNA was removed by treatment with RNase-free DNase I. Biotin-labeled RNA was incubated with streptavidin agarose beads (Invitrogen) overnight after recovery secondary structures. The fresh cell lysates or purified proteins were incubated with RNA-captured beads at 4°C for 1 h. After 5 times washes, the proteins were detected by WB.

### 4.8. Cell Fractionation

Cell fractionation was performed by following our previous protocols [46]. Briefly, cytoplasmic fraction was obtained by cytoplasm lysis buffer after centrifugation at 16,000g for 10 min at 4°C. The nuclear fraction buffer was then added to isolate nuclear fraction in the pellet.

### 4.9. Luciferase Reporter Assay

After the indicated treatment, cells were lysed and the luciferase activities were estimated by using the Dual-Luciferase Reporter Assay kit (Promega, #E1910). The relative luciferase activities were normalized with the value of Renilla.

### 4.10. Measurement of PK and LDH Activities

PK and LDH activities were measured by using the Pyruvate Kinase Test Kit (Comin, PK-1-Y) and LDH activity Test Kit (Solarbio, BC0685), respectively, according to the manufacturer's instructions. In brief, fresh cells were lysed in the lysis buffer provided by the manufacturers. After sonicating, cell lysates were centrifuged at 8,000g for 10 min at 4°C to obtain the supernatants for the assays. PK activity was determined through a LDH-coupled assay with or without the addition of FBP, by monitoring the difference absorbance of NADH within 20 s to 2 min and 20 s at 340 nm, at 37°C. LDH catalyzes the lactate to pyruvate. The latter further converts to pyruvate dinitrophenylhydrazone that exhibits brownish red in alkaline solution, the OD value of which can be determined calorimetrically at 450 nm that is positively correlated with pyruvate concentration and also an indicator of LDH activity. The obtained PK and LDH activities were calculated after normalization to protein concentrations. The values were then normalized to the average of the untreated controls.

### 4.11. Determination of Lactate Production and Glucose Uptake

Lactate production and glucose update were determined by using a lactate assay kit (Njjcbio, A019-2-1) and glucose assay kit (Applygen, E1010-200), respectively, by following the manufacturer's instructions. Briefly, the same numbers of cells were seeded and kept in culture for 48 h. The media were collected and subjected to the indicated assays. Lactate concentration was measured using a NADH-linked enzymatic assay as indicated by the absorbance measured at 530 nm. Glucose uptake was determined using the glucose oxidase method by measuring absorbance at 550 nm. The lactate production and glucose uptake were calculated after normalization to protein concentrations. The values were then normalized to the average of the untreated controls.

### 4.12. Determination of Pyruvate Concentration

Pyruvate concentration was detected using a colorimetric assay (Solarbio, BC2205) according to the manufacturer's instructions. Briefly, cells were lysed in pyruvate extracting buffer provided by the manufacturer. The lysate was mixed with the detection reagent for 2 min. The absorbance was measured at 520 nm with a microplate reader. The relative pyruvate concentration was calculated after normalization to protein concentrations.

### 4.13. Purification of Recombinant PKM2 Proteins

GST-tagged full-length and truncated mutant PKM2 proteins from BL21 bacteria were purified by sonication of BL21 cells after incubating with 0.5 mM IPTG for 16 h at 16°C. After centrifugation, cells were lysed in NETN buffer with Protease Inhibitor Cocktail and then incubated with GST beads for 3 h to enrich GST-PKM2 proteins. After three times washes, protein was eluted with GSH buffer. These purified proteins were ready for in vitro assays.

### 4.14. Measurement of ECAR

ECAR was detected using the XF24 Flux Assay Kit (Seahorse 102340-001) with XF24 Extracellular Flux Analyzer (Seahorse Bioscience). Briefly, cells were plated at 5 × 10^4^ cells per well in a XF24 Cell Culture Microplate (Seahorse 100777-004) cultured at 37°C in 500 *μ*l base medium supplemented with glutamine in a CO_2_-free incubator for 1 h. At this period, a sensor cartridge with glucose (10 mM), oligomycin (1 *μ*M), and 2-DG (50 mM) compound was loaded into a seahorse XF24 analyzer sequentially. When the instrument was ready after loading with the sensor cartridge, the microplate was loaded according to the program prompt. Glycolytic capacity was calculated by Wave Software as the difference between the ECAR following the injection of oligomycin and the basal ECAR reading.

### 4.15. Glutaraldehyde Crosslink Assay

Total protein was lysed in PBS (with 0.1% Triton X-100). 4 *μ*g protein was crosslinked with 0.025% glutaraldehyde for 2 min at 37°C, and the reaction was terminated with Tris-HCl (pH = 8.0, 50 mM). Both uncrosslink control and crosslink proteins were denatured with SDS-loading buffer for 5 min. Then, the same amount of protein was analyzed by WB using an anti-PKM2 antibody (Proteintech, 15822-1-AP).

### 4.16. BrdU Incorporation Assay

The BrdU incorporation was performed following the protocol from Cell Signaling Technology. Briefly, cells were incubated with 0.03 mg/ml final concentration BrdU at 37°C for 30 min. After fixation with 70% cold ethanol at room temperature, cells were treated with 1.5 M HCl for 30 min. Finally, the cells were immunostained with an anti-BrdU antibody, and 500 cells in total from 10 random sights were calculated to determine the BrdU-positive rate.

### 4.17. Trypan Blue Assay

The trypan blue staining assay was performed as previously reported. Briefly, after glucose starvation or treatment with 2-DG, both dead and live cells were collected and stained with the trypan blue solution. Afterward, the death rate of the 500 cells was calculated.

### 4.18. Caspase-3/7 Activity Assay

After indicated treatments, cells were collected to detect caspase-3/7 activity with the Caspase-Glo_3/7 Assay kit (Promega, #G8091) according to the manufacturer's instruction, and each group was conducted in triplicate.

### 4.19. Cell Viability Assay

Cell viability was evaluated with the colorimetric MTT [3-(4,5-dimethylthiazol-2-yl)-2,5-diphenyltetrazolium bromide] assay. After 4-hour incubation, the formazan was dissolved by DMSO and the absorbance was measured at 490 nm.

### 4.20. Single-Cell Suspensions from Tumor Xenografts

Tumors of xenografts were dissociated with surgical scissors into small pieces and digested with DMEM medium with 10% FBS containing 2 mg/ml collagenase I, 100 *μ*g/ml hyaluronidase, and 2 U DNase I for 1 h in a 37°C incubator. Afterward, the cell suspensions were filtered using mesh and washed with PBS. Cells were lysed with ACK lysing buffer to remove red blood cells and washed with PBS once. The cells were kept on ice for other analyses.

### 4.21. Tumor Xenografts

A tumor xenograft assay was conducted by following the previous report [46]. Briefly, the 1 × 10^7^ cells were inoculated into the same female nude mouse (4 and 5 weeks old, Beijing HFK Bioscience Co., Ltd.) subcutaneously. The tumor volumes were monitored every week and calculated as length × width^2^ × 0.5 for 4 weeks. Then, the tumor was dissected, photographed, and weighed. All animal procedures were performed according to the Chinese government published rules for animal experiments (Beijing, China) and approved by the Research Ethics Committee of Harbin Institute of Technology, China.

### 4.22. Flow Cytometry Analysis of THP-1 Cell Surface Markers

THP-1 cells differentiated with 100 ng/mL PMA for 24 h following additional 48 h incubation with the indicated CM or cells obtained from tumor xenografts were collected and fixed with 4% paraformaldehyde for 20 min at 4°C. Then, cell surface expression of CD206 and CD86 was determined by staining cells with the corresponding antibodies (anti-CD206 (1 : 100) and anti-CD86 (1 : 100)) for 1 h at room temperature. After incubation with the second antibodies (1 : 400) for 30 min, samples were analyzed by flow cytometry.

### 4.23. Transwell Assay

1 × 10^5^ THP-1 cells grown on the insert were activated by incubating with PMA (100 ng/ml) [47]. 24 h later, the medium was replaced with fresh medium (without FBS). 600 *μ*L CM from the indicated cell cultures was added in the lower chamber. Cell infiltration was evaluated by the ability of cell migration to the opposite side of the insert. Cells on the lower side of the insert were fixed with 70% cold ethanol and stained with 0.2% crystal violet solution. The images at five random fields were captured, and the relative infiltration rate of THP-1 cells was analyzed by using ImageJ software.

### 4.24. Statistical Analysis

Statistical analysis was done by using GraphPad software, version 5. Data are presented as the means ± standard error of the means (SEM) or standard deviation (SD). Student's *t*-test was applied to assess the statistical significance. Correlations were calculated according to Spearman or Pearson correlation. *P* value < 0.05 was considered significant.

## Data Availability

All data are available in the manuscript, in supplementary materials.

## Supplementary Materials

Supplementary Materials Figure S1: HITT inhibits aerobic glycolysis. Figure S2: HITT inhibits glycolysis by inhibiting PK activity. Figure S3: HITT has no obvious impact on PKM2 expression. Figure S4: HITT and miR-106 are negatively correlated in human clinical samples. Figure S5: HITT-inhibited lactate production had no obvious impact on macrophage infiltration.
