# Supplementary material for: lncRNA HITT Inhibits Lactate Production by Repressing PKM2 Oligomerization to Reduce Tumor Growth and Macrophage Polarization
Source: Research (Wash D C). 2022 Jul 5;2022:9854904. doi: 10.34133/2022/9854904 (PMC9285634; doi:10.34133/2022/9854904)
Supplement: Supplementary Materials — Figure S1: HITT inhibits aerobic glycolysis. Figure S2: HITT inhibits glycolysis by inhibiting PK activity. Figure S3: HITT has no obvious impact on PKM2 expression. Figure S4: HITT and miR-106 are negatively correlated in human clinical samples. Figure S5: HITT-inhibited lactate production had no obvious impact on macrophage infiltration. [file 9854904.f1.docx]

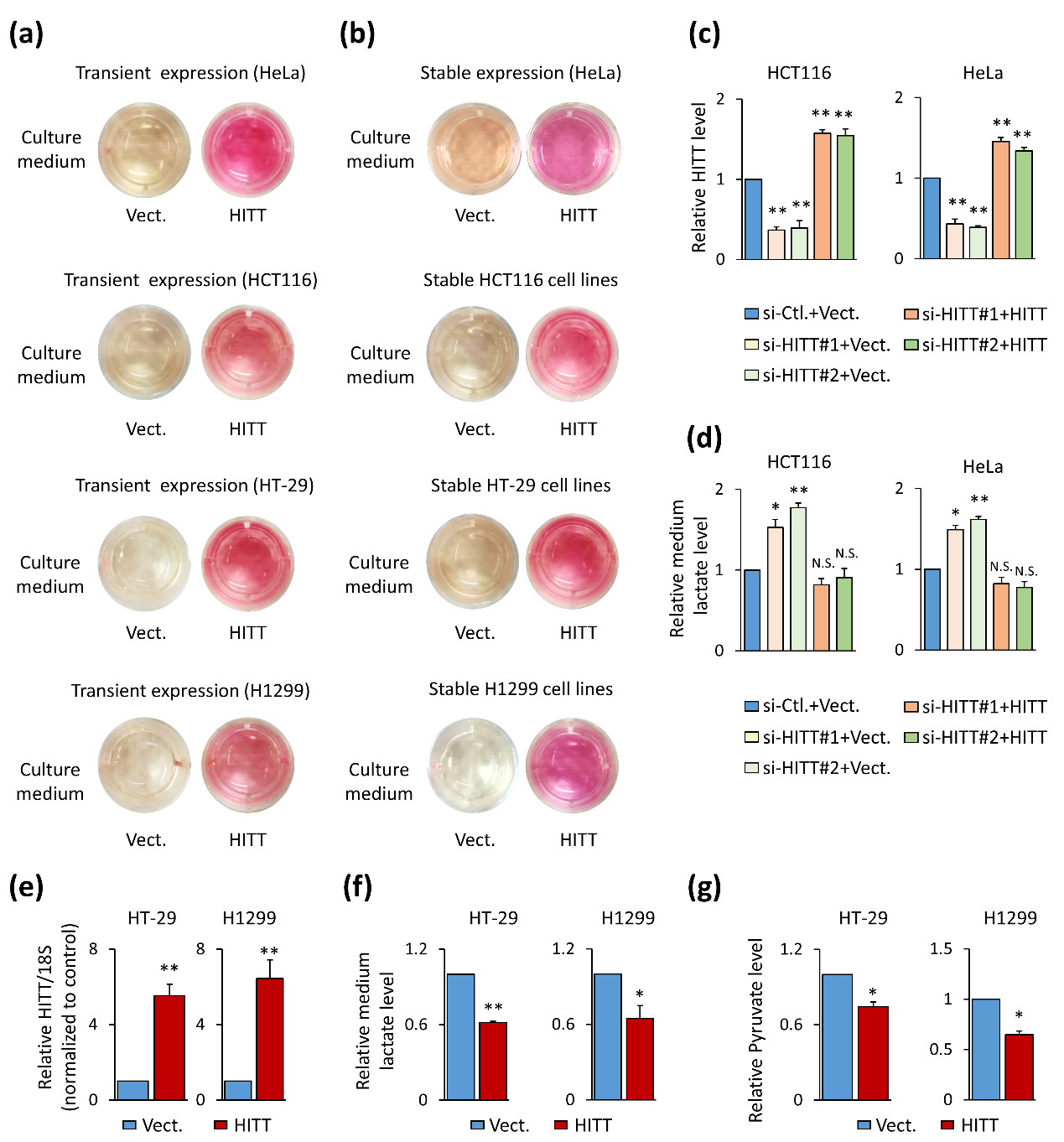


**Figure S1 HITT inhibits aerobic glycolysis**

(a and b) Representative images of the media colors of transient (a) or stable (b) HITT-overexpressing HeLa, HCT116, HT-29, and H1299 cells. (c and d) HITT (c) or lactic acid (d) level of HITT KD were determined after transfected with or without HITT overexpression HCT116 (left) and HeLa (right) cells. (e) The overexpression efficiencies of HITT in HT-29 (left) and H1299 (right) cells were determined by real-time RT-PCR. (f and g) Lactate production in culture medium (f) and pyruvate levels (g) of HT-29 (left) and H1299 (right) cells were analyzed using a Lactate Assay kit. Data are derived from three independent experiments and presented as mean ± SEM in the bar graphs. **P* < 0.05; ***P* < 0.01; N.S., not significant (c-g). Vect., vector; Ctl., control.

**
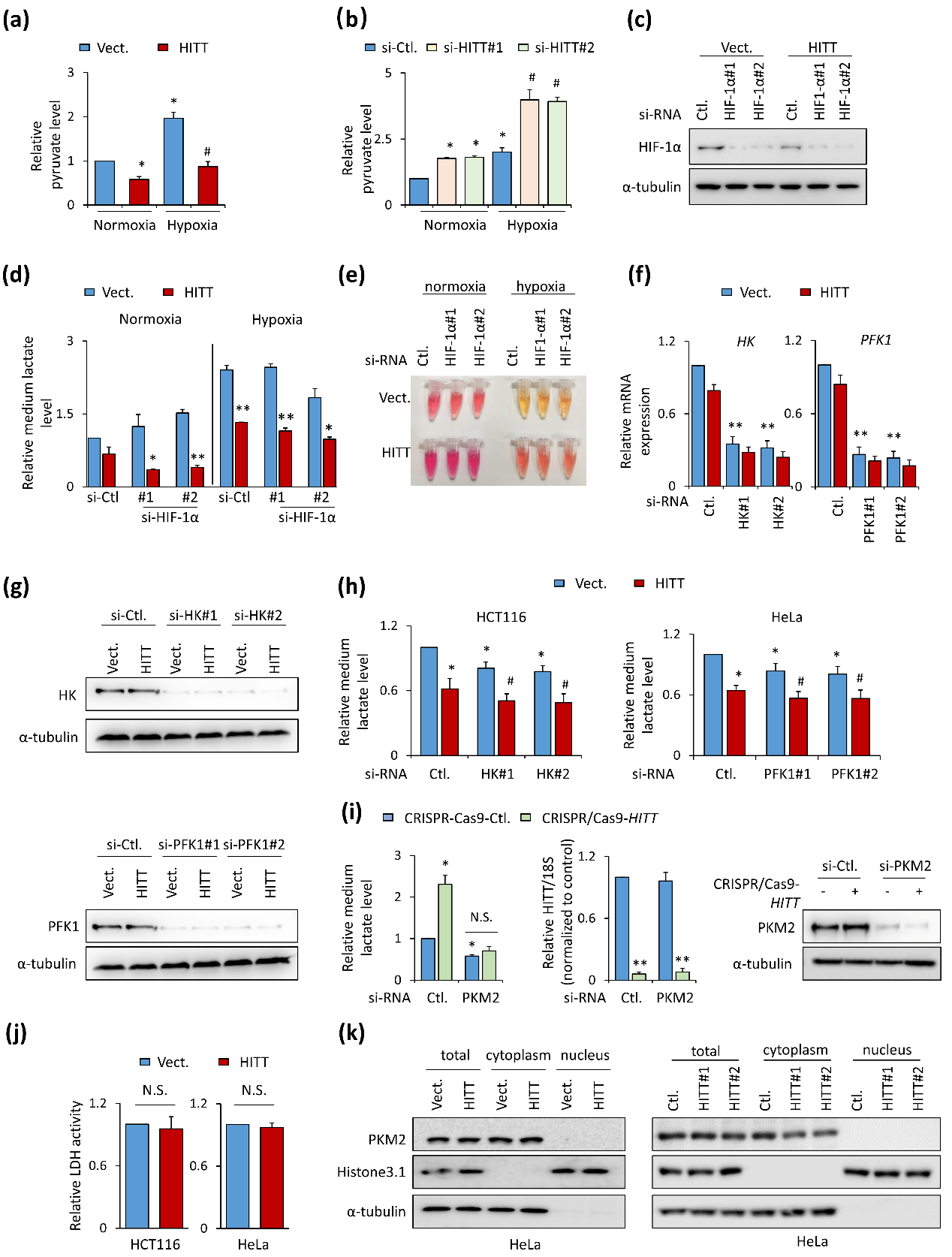
**

**Figure S2 HITT inhibits glycolysis by inhibiting PK activity**

(a and b) The pyruvate production of HITT-overexpressing (a) or KD (b) cells were determined under normoxia or hypoxia. (c) The efficiencies of two independent oligos-mediated HIF-1α KD in the control and HITT stable expressing cells were determined by WB. (d and e) lactate concentrations (d) of the culture media of HITT stable expressing HeLa cells after siRNA-mediated HIF-1α KD under normoxia or hypoxia were determined as described in the Materials and Methods, representative color images of the corresponding culture media are presented (e). (f and g) The KD efficiencies of HK and PFK1 were confirmed by qRT-PCR and WB. (h), lactate production in culture media was determined after the indicated treatments in HeLa cells. (i) The lactate productions were analyzed in the culture medium of HeLa cells transfected with CRISPR/Cas9-*HITT* plasmid with or without PKM2 KD (left). The KD efficiencies of HITT and PKM2 were determined by qRT-PCR (middle) and WB (right) (j) LDH activities were compared in HITT-overexpressing and control HCT116 and HeLa cells. (k) Cytoplasmic and nuclear PKM2 levels were detected after cell fractionation in HITT-overexpressing (left) or KD (right) HeLa cells. Data are derived from three independent experiments and presented as mean ± SEM in the bar graphs. Values of controls were normalized to 1. **P* < 0.05; ***P* < 0.01; N.S., not significant (a, b, d, f and h-j); ^#^*P* < 0.05, compared with the Vect. group treated with indicated vector or siRNAs (a and h). Vect., vector; Ctl., control.


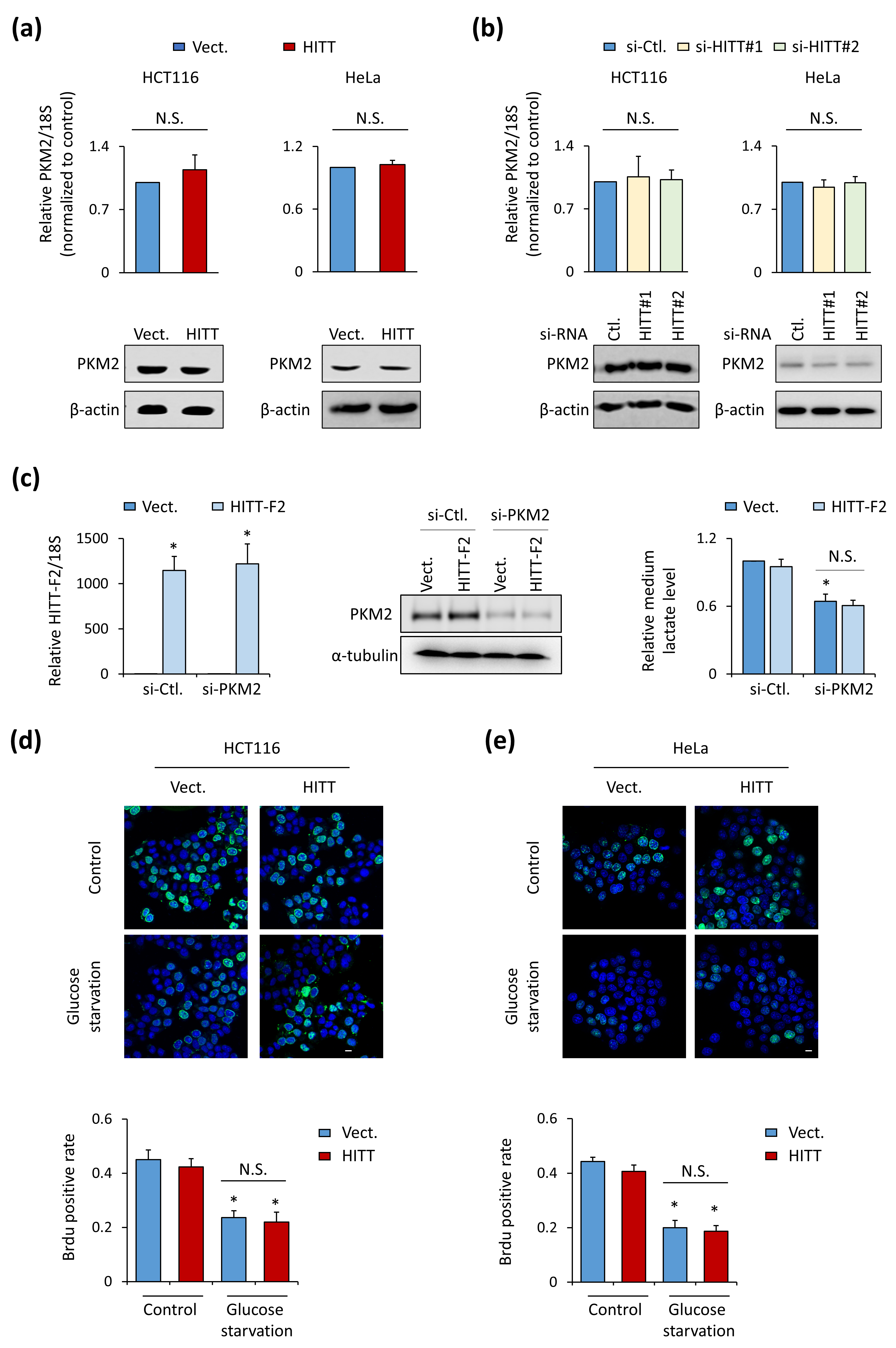


**Figure S3 HITT has no obvious impact on PKM2 expression**

(a and b) PKM2 mRNA (top) and protein levels (bottom) were detected by real-time RT-PCR and WB, respectively, in HITT-overexpressing (a) or KD (b) HCT116 and HeLa cells. (c) Lactate productions of HITT F2 overexpression HCT116 cells were detected after siRNA-mediated PKM2 (middle). The overexpression efficiency of HITT F2 fragment and the KD efficiency of PKM2 were determined by qRT-PCR (left) and WB (right), respectively. (d and e) The HITT-overexpressing HCT116 (d) and HeLa (e) cells proliferation were analyzed by Brdu staining. Scale bar, 10μm. Values of controls were normalized to 1. **P* < 0.05; ***P* < 0.01; N.S., not significant (a-d). Vect., vector; Ctl., control.


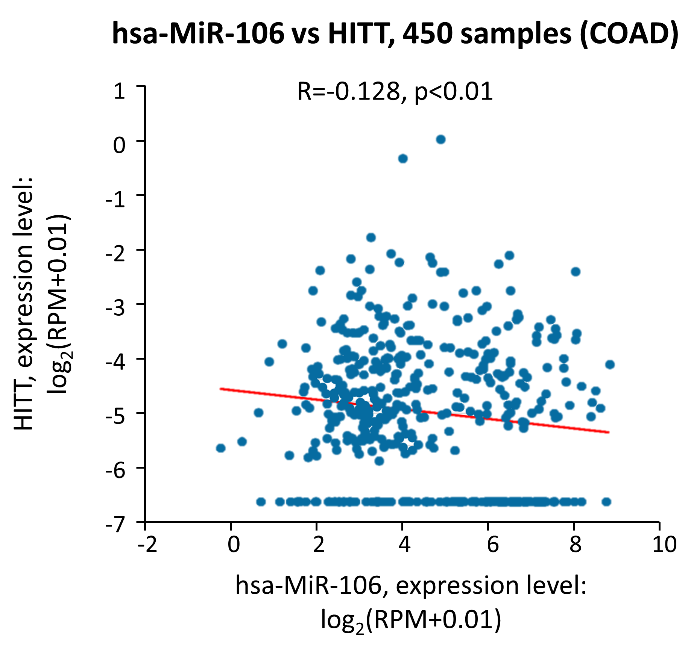


**Figure S4** **HITT and MiR-106 are negatively correlated in human clinical samples**

Correlation analysis of HITT and MiR-106 in clinical colon adenocarcinoma was determined by analysis the data derived from the ENCORI Pan-Cancer Analysis Platform (https://starbase.sysu.edu.cn/panCancer.php).

**
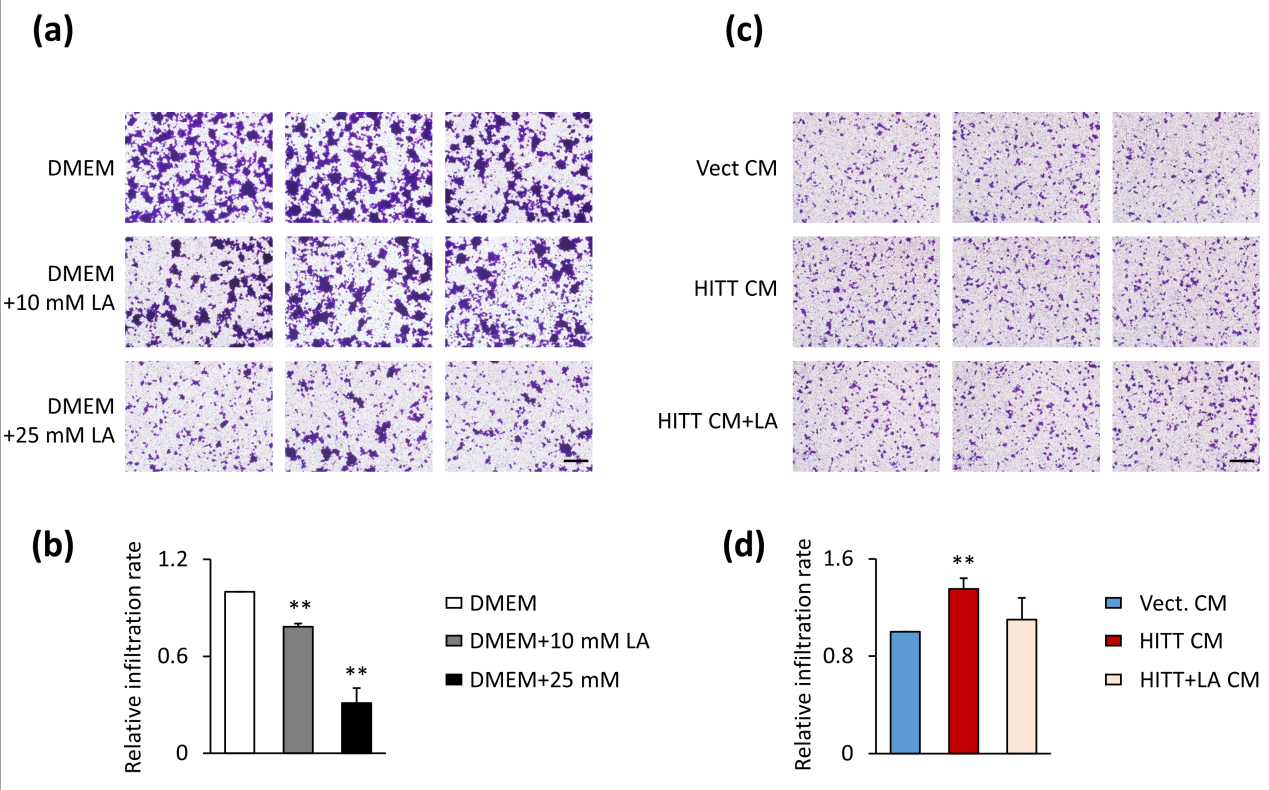
**

**Figure S5 HITT-inhibited lactate production had no obvious impact on macrophage infiltration**

(a and b) THP-1 cell infiltration was evaluated by transwell assay after adding 10mM or 25mM lactate in the lower chamber (a), quantification of three independent experiments is shown in the bar graph (b). (c and d) THP-1 cell infiltration was evaluated by transwell assay with the indicated CM in the lower chamber (c), quantification from three independent experiments is shown in the bar graph (d). Scale bar, 100μm. Data are derived from three independent experiments and presented as mean ± SEM in the bar graphs. **P* < 0.05; ***P* < 0.01 (b and d). Vect., vector; LA, lactic acid.
